# A survey of student loan burden among United States Chiropractors: Insights on debt, relief, and educational value

**DOI:** 10.1371/journal.pone.0347127

**Published:** 2026-04-13

**Authors:** Samuel M. Schut, Zachary A. Cupler, Brian C. Coleman

**Affiliations:** 1 Department of Wellness and Preventive Medicine, Cleveland Clinic, Cleveland, Ohio, United States of America; 2 Center for Wellness Research and Training, Cleveland Clinic, Cleveland, Ohio, United States of America; 3 Cleveland Clinic Lerner College of Medicine of Case Western Reserve University School of Medicine, Cleveland, Ohio, United States of America; 4 Physical Medicine and Rehabilitative Services, Butler VA Healthcare System, Butler, Pennsylvania, United States of America; 5 Department of Emergency Medicine, Yale School of Medicine, New Haven, Connecticut, United States of America; 6 Department of Biomedical Informatics and Data Science, Yale School of Medicine, New Haven, Connecticut, United States of America; 7 Department of Biostatistics, Yale School of Public Health, New Haven, Connecticut, United States of America; 8 Pain Research, Informatics, Multimorbidities and Education (PRIME) Center, VA Connecticut Healthcare System, West Haven, Connecticut, United States of America; Covenant University, NIGERIA

## Abstract

**Background:**

Although student loans have made health professional education accessible for a greater proportion of the US population, rising student indebtedness has incited considerable discourse. This study aimed to characterize US chiropractors’ self-reported student loan debt, student loan relief opportunities, and perceived value of chiropractic training.

**Methods:**

A 38-item cross-sectional, anonymous, electronic survey was developed. Chiropractors who graduated from a US-based, Council on Chiropractic Education-accredited Doctor of Chiropractic program (DCP) were recruited. The survey was conducted between February 2025 to March 2025. Survey domains included (1) demographics, (2) financial characteristics, (3) loan relief, and (4) educational and career value. Descriptive statistics and visualizations were used for analysis.

**Results:**

A total of 1,455 responses were collected. The mean (SD) and median (Q1-Q3) student loan debt at graduation was $176,297 ($89,460) and $185,000 ($120,000–$240,000). At survey completion, 85% retained student loan debt, averaging $232,062 ($102,691) with a median of $240,000 ($177,500–$290,000). Mean (SD) gross income in 2023 was $99,068 ($100,349); median (Q1-Q3) was $76,000 ($50,000-$115,000). Approximately 87% of respondents reported being ineligible or unsure about eligibility for loan relief programs. Over half (53.3%) disagreed or strongly disagreed that their DCP provided a positive return on investment (ROI), and approximately 70% rated the financial ROI of DCP training as low or very low. Perceptions of non-financial ROI were more favorable. Overall, 65% would not choose a chiropractic career again; among them, 67.3% would pursue a career in another healthcare field.

**Conclusions:**

US chiropractors are burdened with considerable student loan debt that outpaces gross income. The findings of this study are commensurate with prior studies investigating chiropractic educational debt, yet likely are not unique to the profession and represent larger challenges faced by many modern US health professions. Innovation is likely needed to support the sustainability of the chiropractic profession given tension between educational debt and income.

## Introduction

Student loans provide crucial financial support for many individuals pursuing higher education in the United States (US), helping to cover educational costs including tuition, class materials, and associated living expenses. Although student loans have made postsecondary education more accessible for a greater proportion of the US population, thus accomplishing one of its original goals, rising student indebtedness has incited considerable discourse over affordability, cancellation policies, and the erosion of value for borrowers’ degrees.[1–5] Some health professional training programs, including dentistry, medicine, optometry, physical therapy, and pharmacy, have investigated graduating students’ educational debt, repayment strategies, debt to income ratios, and planned participation in loan relief programs.[6–11] In contrast, the US chiropractic profession has yet to publish large-scale reports on graduates’ student loan characteristics.

Graduates of health professional training programs, particularly allied health disciplines, frequently accumulate sizeable educational debt.[11] In 2020, over 90% of health science professional practice program (i.e., chiropractic, dentistry, optometry, pharmacy, podiatry, and veterinary medicine, but excluding medicine) graduates had student loans, with an average cumulative amount per borrower of $258,120 for undergraduate and graduate education combined.[12] Considered in isolation, high student loan balances are not automatically alarming when income comfortably offsets debt. However, external factors including declining reimbursement, weak labor market outcomes, and difficulty accessing relief programs create relevant challenges.[11,13] These obstacles are compounded further in professional settings (e.g., sole proprietorships, independent contracting) which can produce fluctuating income over a period of planned debt repayment. To accurately assess overall measures and impacts of financial performance in the chiropractic profession and inform strategies to address potential shortcomings, it is first necessary to understand the characteristics of student loan burden and income among DCs in the US.

To date, there has been sparse characterization of chiropractic educational debt, often being collected alongside other health professions. Preliminary reporting by Mirtz et al. found student loan debt a factor for a majority (64%) of non-practicing DC’s perception of career success.[14] A 2014 survey of 57 chiropractic students at one US DCP reported most respondents (74%) estimated that they would accumulate over $125,000 in student loan debt by graduation.[15] More recently, a 2024 study characterized self-reported data of US chiropractic graduates (*n = 448*) from a student loan consulting firm, finding that the average student loan debt for DCs was high, with a mean and median student loan debt of $249,149 and $240,000, respectively.[16] Of this cross-section, 80% of DCs held between $150,000–$349,999 in student loan debt.[16] However, individuals who seek debt counseling may possess different loan characteristics and may cope with repayment in different ways than the rest of borrowers, suggesting a potentially false impression of the broader chiropractic populace.

Although the chiropractic profession has historically been isolated (i.e., training and workplace) from allopathic medicine and other health professions, in recent decades the profession has been shown to contribute substantial value to the US healthcare system in guideline concordant musculoskeletal pain management. Patients frequently seek chiropractic care, especially for spinal pain concerns, with large cohort studies of commercial and Medicare Advantage insurance enrollees finding DCs as the most common initiating provider for neck pain (45%) and second most common initiating provider for low back pain (23%, after primary care providers).[17,18] Chiropractor’s treatment approaches commonly comprise guideline concordant recommendations for the management of spinal pain. In addition to trial evidence demonstrating small effects on pain intensity and pain-related function, chiropractic care serves as an important nonpharmacologic pain management care pathway, contributing to opioid-sparing strategies and helping to reduce reliance on pharmacologic therapies.[19–22]

Considering these current breaches in knowledge and added societal value of chiropractic in the greater healthcare landscape, a broader and more generalizable understanding of US DCs’ self-reported student loan debt may accurately frame student debt amounts relative to earning potential, while transparently informing accreditors, policy makers, DCP leadership, and prospective DCP students to ensure the profession’s financial sustainability. The objective of this study is to characterize US chiropractors’ self-reported student loan debt, student loan relief opportunities, and perceived value of chiropractic training.

## Methods

### Study design, setting, and ethics

We conducted an online, cross-sectional, anonymous survey from February 18, 2025 to March 31, 2025 consistent with recommended frameworks for survey research in health professions.[23–26] We adhered to The Checklist for Reporting of Survey Studies for reporting (S1 Appendix).[27] Ethical approval for this exempt study was provided by the Yale University Institutional Review Board (#2000039247).

### Participants and context

The target population for participation was DCs who graduated from a US-based, CCE-USA accredited DCP. Current DCP students and individuals who had never attended a CCE-USA accredited DCP or had attended a non-US program were excluded. State license status and current occupation did not limit respondents’ eligibility to participate. The survey was conducted in English.

### Survey development

The novel survey instrument was developed through an iterative process. We anchored the survey in a conceptual framework for chiropractic student loan debt following an “*Input* → *Mechanism* → *Output*” logic model, where the central “*Mechanism*” is accumulation and persistence of student loan debt for DCs, upstream factors contribute as inputs, and downstream manifestations follow as outputs. This framework anchored our descriptive inquiry, allowing us to first characterize the immediate burden “*Mechanism*” as a prerequisite for future, multi-dimensional assessments of the systemic inputs and socio-economic outputs of debt burden.

Key topics and questions were formulated based on a review of published surveys of other healthcare professional disciplines, annual health professional trainee reports, and germane sources in the gray literature.[28–33] Existing items were then adapted to suit the context of US DCs and the objectives of the study. Face and content validity were addressed with pilot testing among a group of 10 DCs who ranged from recently graduated to 14 years post-graduation and worked in a variety of settings including private practice, the Veterans Health Administration, private hospital systems, or were attending advanced post-graduate training programs. Following pilot testing, we enhanced survey item clarity and interoperable display across mobile and desktop platforms. The approximate time required to complete the survey was 10 minutes.

The final survey instrument consisted of four domains and 38 possible items (S2 Appendix). Responses were elicited via single- or multiple-choice questions and free-text numeric entries with associated response validation. Skip logic, branching, and action tags were employed throughout the survey to help reduce irrelevant responses, reduce time burden, and enhance data accuracy.

The first domain collected demographic information and practice characteristics including age, sex, race/ethnicity, years since graduation from a DCP, practice status, area of primary practice, additional degrees, and DCP granting DC degree. Participants were also queried about their perceived best role of DCs in the health care system – spine/neuromusculoskeletal (NMSK-focused) care, general primary care, or vertebral subluxation detection and removal (subluxation-focused) – based off prior work by Gliedt et al.[34] The second domain, financial characteristics, included 2023 gross income, total student loan debt both upon graduation from DCP and at the time of survey completion, loan statuses, monthly payments, loan types, interest rates, time in repayment, and repayment plans. The third domain focused on student loan relief programs and included eligibility for relief programs, progress towards forgiveness, and whether respondents sought employment based upon relief programs. The last domain assessed perceived educational and career value using Likert scale responses and included likelihood to recommend a chiropractic career, financial value of DCP education, and likelihood to pursue a chiropractic career again. Finally, participants were asked to estimate the perceived total cost of attendance (including tuition, fees, class supplies, and living expenses) for a DCP in 2025.

### Survey administration, sampling strategy, and data management

Web-based, electronic convenience sampling was implemented to maximize catchment. Primary efforts to disseminate the survey link focused on the constituent membership of the two US national chiropractic associations – the American Chiropractic Association (ACA) and the International Chiropractic Association (ICA).[35,36] While exact membership counts are not reported publicly, the ACA and ICA collectively represent the US chiropractic profession and jointly interface with a large proportion of US DCs.[37] Second, we approached the CCE-USA and each US-based accredited DCPs’ alumni office (or equivalent) to request each consider voluntary electronic distribution of the survey link among their professional networks and alumni listservs.[38] Third, we posted the electronic survey link to two active social media (Facebook) groups for chiropractors with large US DC bases: *Forward Thinking Chiropractic Alliance* and *Evidence-Based Chiropractors Network*.

The survey remained open for 6 weeks, and the study team sent e-mails to ACA, ICA, and DCP alumni office staff to promote and socialize the electronic survey link to their respective active membership and alumni contact lists at least twice during the survey period. No restrictions were placed on ACA, ICA, DCP institutions, or individual DCs’ ability to socialize the survey through their respective social media accounts, online closed community platforms, or through electronic correspondence (e.g., text or email) with colleagues, enabling snowball sampling. Social media posts to the referenced groups occurred at three timepoints during the survey period. A survey invitation reach denominator was incalculable for this study given the open and multi-pronged recruitment approach, rendering calculation of an accurate response rate as impractical. An estimated participation rate was thus calculated as the ratio of survey completion (full or partial) to survey link clicks as a proxy measure of intention to complete the survey.

Participation was voluntary, withdrawal was allowed at any time, and written consent was obtained at the start of the survey. There was no remuneration or incentive for participating. Given the potentially sensitive nature of the information requested, participants had the option to opt out of any item that they preferred not to answer. All data was collected anonymously, with no unique identifiers captured (including no collection of internet protocol addresses). Survey responses were collected and managed using Research Electronic Data Capture (REDCap) hosted at Yale University.[39,40] Access to REDCap and all individual-level response data was secured through multi-factor authentication and enforced via institutional single sign-on protocols, ensuring compliance with institutional policies and the approved study protocol for the entirety of the study.

### Cost of attendance estimates

We used publicly available 2024−25 academic year cost of attendance (COA) estimates to derive a total COA projection by multiplying term estimates by program length for each CCE-USA accredited, US-based DCP (S3 Appendix). Of 17 total DCPs, 4 were excluded since they did not report estimates for three or more categories of expenses (e.g., housing/food, transportation, books/supplies/equipment), precluding an accurate estimate of total COA. The excluded programs did not appear to systematically differ from those that reported COA estimates. Individual DCP estimates were averaged to determine an overall national estimate.

### Statistical analysis

All statistical analyses were completed in R Studio (Boston, MA) using R version 4.4.0 (R Core Team, Vienna, Austria).[41] Descriptive statistics were generated for demographics, financial characteristics, relief program eligibility/participation, and perceptions of educational and career value. This was an exploratory and descriptive analysis of survey responses, thus formal hypothesis testing was beyond the scope of this work. Where of interest, responses were stratified based on chiropractic professional subgroups. While exploratory, we suspected generally more positive perceptions of chiropractic career satisfaction and educational value among the subluxation-focused subgroup compared to the NMSK and primary care subgroups. Comparative observations between subgroups were intended for hypothesis generation and not interpretation as evidence of statistically significant differences in the general population.

Missing data for relevant variables were quantified and reported with other summary statistics, with no imputation performed. We corrected responses for years since graduation that clearly indicated a year (e.g., “2015”) as the difference between 2025 and the reported value. We also excluded responses for estimated 2024−25 COA that were less than $1,000 as unrealistic. Outlier responses were addressed using a hybrid approach consistent with best practices for outlier identification and handling to discern erroneous values from valid but extreme values [42,43]. This involved statistical testing using Rosner's generalized extreme Studentized deviate (GESD) test, normality assessed visually using Q-Q plots, and study team’s subject-matter expert review to exclude clearly erroneous values for reported debt at graduation and survey completion. While we tested for outliers in reported 2023 gross income using the same hybrid outlier detection approach, we did not exclude any values as the identified outlier threshold ($300,000) remained reasonable. There were very few values identified in a long right-tail of the distribution ranging to $1.2M; however, we suspect more erroneous values were entered in the lower range (e.g., $18) that were not identified using the statistical methods. A validation step was applied comparing measures of central tendency and distributions before and after outlier exclusion to observe for internal consistency between the raw and outlier-filtered data.

General trends in several key financial metrics were summarized through data visualization. We visualized the mean values of debt at graduation, debt at survey completion, reported gross income (for the 2023 tax year), and estimated total COA, collectively plotted by years since graduation. A linear regression line was superimposed to depict the overall trend in these average values without weighting for the number of responses at each year since graduation. Additionally, a raincloud plot was used to summarize the distribution of responses for debt at survey completion, reported gross income, monthly payment, and interest rate as key interrelated factors in student loan repayment planning. To ensure robust analysis, only data from respondents indicating 0–25 years since graduation were included in all visualizations, as observations beyond this range demonstrated less stability due to much smaller sample sizes (range n = 0 to n = 13 for 26–51 years since graduation).

## Results

There were 1,480 complete or partial engagements with the survey. Following confirmation of eligibility and consent to participate (n = 25 ineligible and/or not consenting to participation), 1,455 responses were retained for analysis. Given 3,928 survey link clicks, this represented a 37.7% participation rate from the hyperlink point of access.

### Demographics

The mean (SD) age of participants was 38.9 (9.42) years, 54.9% were female, 83.8% were non-Hispanic White, 98.8% were currently or had practiced chiropractic in the US, and the mean (SD) number of years since graduating from a DCP was 11.2 (8.62) (Table 1). Twenty-one distinct DCPs were represented among respondents, with a maximum of 245 respondents from a single institution. Most respondents possessed a bachelor’s degree (71.1%), while few attained a master’s or other graduate degree (13.7%) or other doctoral or professional degree (5.1%). Eighty percent of bachelor’s degrees were obtained by respondents prior to matriculating into a DCP; in contrast, most master’s or other graduate degrees were completed while concurrently enrolled in a DCP (41.5%) (S4 Appendix).

**Table 1:** Respondent demographics (N = 1,455).

Variable	
Age (years) (n = 1,359)	
Mean (SD)	38.9 (9.42)
Median (Q1-Q3)	38 (32-44)
Min, Max	21, 78
Sex (n = 1,357), n (%)	
Female	745 (54.9%)
Male	612 (45.1%)
Race/ethnicity (n = 1,349), n (%)	
non-Hispanic White	1,131 (83.8%)
Hispanic	65 (4.8%)
non-Hispanic Black	16 (1.2%)
Multiracial	25 (1.9%)
non-Hispanic Asian	22 (1.6%)
Other	90 (6.7%)
Unique DC institutions^a^	21
Participants by institution (Min, Max)	(2, 245)
Years since DCP graduation (n = 1,365)	
Mean (SD)	11.2 (8.62)
Median (Q1-Q3)	10 (5-15)
Min, Max	0, 51
Practicing chiropractic in US (n = 1,324), n (%)	
Currently practicing	1,227 (92.7%)
Previously practiced	81 (6.1%)
Never practiced	16 (1.2%)
Area of primary work (n = 1,354), n (%)	
Clinical practice	1,216 (89.8%)
Education	46 (3.4%)
Administrative	20 (1.5%)
Research	19 (1.4%)
Retired/Other	53 (3.9%)
Clinical practice setting^b^ (n = 1,455), n (%)	
Solo practice	598 (41.1%)
Group practice (DCs only)	468 (32.2%)
Multi-disciplinary practice (DCs and non-DC clinicians)	241 (16.6%)
Franchised practice	76 (5.2%)
VA/DOD	66 (4.5%)
Hospital system (non-VA/DOD)	37 (2.5%)
Other academic healthcare setting	36 (1.8%)
Post-graduate residency or fellowship	12 (0.8%)
Other	15 (1.0%)
Never in clinical practice	8 (0.5%)
Best role for DCs in healthcare (n = 1,347), n (%)	
Spine or NMSK care	1030 (76.5%)
Subluxation detection and removal	208 (15.4%)
General primary care	109 (8.1%)

SD: Standard deviation; Q1-Q3: 1^st^ quartile-3^rd^ quartile; DC: Doctor of Chiropractic; DCP: Doctor of Chiropractic program; US: United States; VA: Veterans Affairs; DOD: Department of Defense; NMSK: neuromusculoskeletal; ^a^Includes closed DCPs as separate programs; ^b^Non-exclusive groups

Clinical practice accounted for most respondents’ area of primary work (89.8%), occurring primarily in solo practice, followed by DC-only group practice, and multidisciplinary practice (DCs and non-DC clinicians). Of those who responded to the item asking about the best role of DCs in the greater healthcare system (n = 1,347), over three-quarters indicated NMSK specialists (76.5%). A minority of these respondents identified subluxation-focus (15.4%) or general primary care (8.1%).

### Financial characteristics

Nearly 90% of respondents reported graduating from a DCP with federal or private student loan debt, including debt from other degrees obtained prior to or during the DCP. At the time of graduation, respondents’ mean (SD) and median (Q1-Q3) student loan debt were $176,297 ($89,460) and $185,000 ($120,000-$240,000), respectively (Table 2). Approximately 85% of respondents retained student loan debt at the time of survey completion, with a mean (SD) of $232,062 ($102,691) and median (Q1-Q3) of $240,000 ($177,500-$290,000). The median (Q1-Q3) interest rate was 6.50% (5.70%−7.00%), with a maximum reported value of 17.50% (Table 2). The mean (SD) and median (Q1-Q3) monthly payment amount of respondent’s loans were $686.90 ($813.22) and $450 ($20-$1,000) (Table 2). Income-driven repayment was the most used repayment plan across all degree types except for associate degrees; for DC degrees specifically, 80.9% reported using an income-driven repayment plan (S5 Appendix). Approximately 60% of respondents were in Saving on a Valuable Education (SAVE) plan administrative forbearance (i.e., temporary, non-interest accruing payment pause) at the time of survey completion. The mean (SD) and median (Q1-Q3) years in repayment on DC degrees among respondents were 8.14 (7.07) and 7.00 (2–13), respectively (S6 Appendix). Mean (SD) and median (Q1-Q3) 2023 gross income was $99,068 ($100,349) and $76,000 ($50,000-$115,000), respectively (Table 2).

**Table 2:** Financial characteristics of respondents (N = 1,455).

Variable	
Gross income in 2023 (n = 1,292)	
Mean (SD)	$99,068 ($100,349)
Median (Q1-Q3)	$76,000 ($50,000-$115,000)
Min, Max	$18, $1.2M
Debt at DCP graduation^a^ (n = 1,292), n (%)	
Yes	1158 (89.6%)
No	134 (10.4%)
Amount owed at graduation (n = 1,141)	
Mean (SD)	$176,297 ($89,460)
Median (Q1-Q3)	$185,000 ($120,000-$240,000)
Min, Max	$100, $460,000
Outliers (Min, Max)^b^	7 ($536,000, $250M)
Debt presently^a^ (n = 1,281), n (%)	
Yes	1082 (84.5%)
No	199 (15.5%)
Amount owed presently (n = 1,067)	
Mean (SD)	$232,062 ($102,691)
Median (Q1-Q3)	$240,000 ($177,500-$290,000)
Min, Max	$291, $627,000
Outliers (Min, Max)^b^	7 ($768,000, $240M)
Interest rate^a^ (n = 1,040)	
Mean (SD)	6.19% (1.60%)
Median (Q1-Q3)	6.50% (5.70%−7.00%)
Min, Max	0.00%, 17.50%
Monthly payment^c^ (n = 1,043)	
Mean (SD)	$686.90 ($813.22)
Median (Q1-Q3)	$450 ($20-$1,000)
Min, Max	$0, $5,300
Administrative forbearance due to SAVE litigation^d^ (n = 1,045), n (%)	
Yes	612 (58.6%)
No	369 (35.3%)
Unsure	64 (6.1%)
Estimated total COA for US chiropractic school^e^ (n = 1,173)	(n = 1,173)
Mean (SD)	$230,000 (72,900)
Median (Q1-Q3)	$225,000 (200,000-250,000)

SD: Standard deviation; Q1-Q3: 1^st^ quartile-3^rd^ quartile; DCP: Doctor of Chiropractic program; SAVE: Saving on a Valuable Education; COA: Cost of attendance; ^a^federal or private, from any level of education; ^b^Outliers excluded from summary statistics; ^c^Values > $10,000 excluded as outliers (n = 3); ^d^At time of survey completion; ^e^Including tuition, fees, class supplies, and living expenses for the entire duration of the program

**Fig 1:**
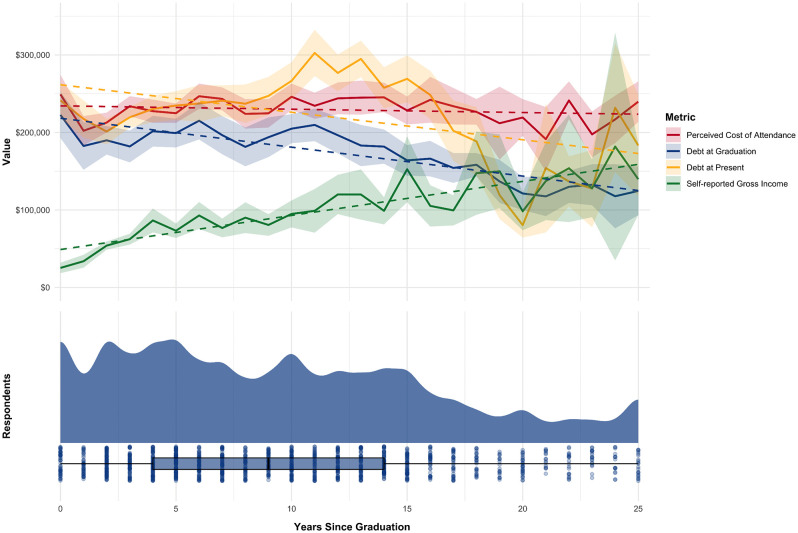
Self-reported financial metrics, averaged by years since graduation.

The averages of relevant financial metrics, grouped by years since graduation and unweighted for sample size, are shown in Fig 1 for respondents within 25 years of graduation. A notable gap exists between average self-reported gross income and average student loan debt at graduation and at survey completion. Average self-reported debt at graduation and at present both show gradual improvements as time since graduation increases, while perceived cost of DCP attendance was generally consistent across all years. In this sample, the average reported income shows general increases over time and reached the $100,000 threshold at the 11-year post-graduation mark; however, this trend should be interpreted with caution given the limited sample size in later career strata.

**Fig 2:**
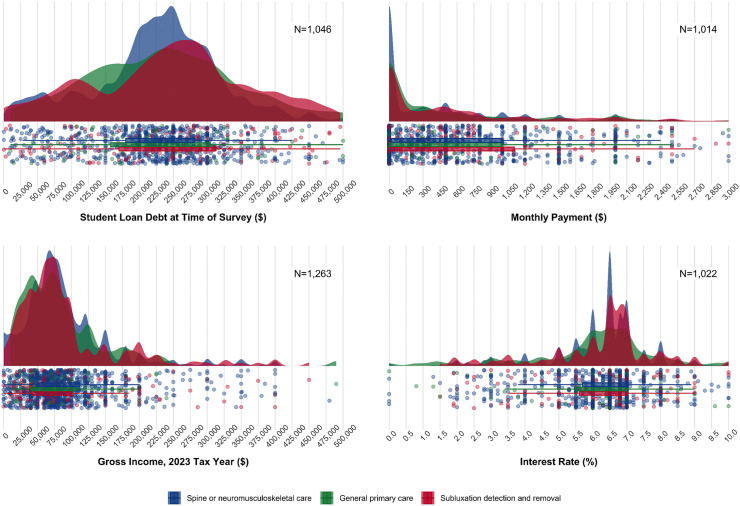
Student loan debt and income metrics distributions by each of three self-identified chiropractic professional subgroups.

Fig 2 presents the distributions of student loan debt at survey completion, monthly payment, income, and interest rate, stratified by self-identified best role of chiropractors in the healthcare system. The distributions were consistent across the defined roles for each inter-related metric.

### Student loan relief program characteristics

Nearly 90% of respondents self-reported either that they were ineligible for loan relief, or were unsure, based on current professional employment (Table 3). Of the few reporting eligibility (n = 158), the majority (87.3%) indicated qualification for the Public Service Loan Forgiveness (PSLF) program. A similar proportion of individuals reported participation, either currently or previously, in student loan relief programs (n = 167), with PSLF being the most common (63.4%). The mean (SD) and median (Q1-Q3) total number of payments made towards PSLF as of survey completion were 54.5 (42.0) and 47.5 (16.5–95.25), respectively. One-third of respondents indicated seeking employment based on student loan relief opportunities.

**Table 3:** Student loan debt relief among respondents (N = 1,455).

Variable	
Eligible for any student loan relief (n = 1,240), n (%)	
Yes	158 (12.7%)
No	967 (78.0%)
Unsure	115 (9.3%)
Eligible relief program type^a^ (n = 158), n (%)	
PSLF Program	138 (87.3%)
NIH Loan Repayment Program	12 (7.6%)
NHSC Loan Repayment Program	0 (0%)
IHS Loan Repayment Program	2 (0.1%)
HRSA Faculty Loan Repayment Program	2 (0.1%)
State-specific loan relief programs	3 (0.2%)
Employer-sponsored loan relief programs	0 (0%)
Sought out employment based on relief eligibility (n = 1,237), n (%)	
Yes	428 (34.6%)
No	809 (65.4%)
Participation in student loan relief (n = 1,236), n (%)	
Yes	167 (13.5%)
No	965 (78.1%)
Unsure	104 (8.4%)
Participation in relief program type^a^ (n = 167), n (%)	
PSLF Program	106 (63.5%)
NIH Loan Repayment Program	3 (0.2%)
NHSC Loan Repayment Program	0 (0%)
IHS Loan Repayment Program	1 (0.1%)
HRSA Faculty Loan Repayment Program	0 (0%)
State-specific loan relief programs	4 (0.3%)
Employer-sponsored loan relief programs	0 (0%)
Total payments towards PSLF^b^ (n = 104)	
Mean (SD)	54.5 (42.0)
Median (Q1-Q3)	47.5 (16.5-95.25)
Min, Max	0, 120
Total qualifying payments towards PSLF^c^ (n = 103)	
Mean (SD)	47.1 (40.6)
Median (Q1-Q3)	36.0 (12.0-75.0)
Min, Max	0, 120

SD: Standard deviation; Q1-Q3: 1^st^ quartile-3^rd^ quartile; PSLF: Public Service Loan Forgiveness; NIH: National Institutes of Health; NHSC: National Health Service Corps; IHS: Indian Health Service; HRSA: Health Resources and Services Administration; ^a^Non-exclusive groups; ^b^Includes both qualifying and payments requiring employer certification, adjusted to max 120; ^c^Includes only payments with employer certification, adjusted to max 120

### Educational and career value characteristics

Overall, 65% of respondents would not pursue a career in chiropractic again, though 67.3% of the dissenting group would pursue a career in another healthcare field (Table 4). Nearly 1 in 5 respondents would pursue a career outside of healthcare, irrespective of the professional subgroup that they identified as the best role for DCs. While a higher percentage of respondents in the subluxation-focused subgroup would pursue a career in chiropractic (46.7%) compared to those in the NMSK (32.7%) or general primary care (32.3%) group, over half would not pursue a career in chiropractic.

**Table 4:** Educational and career value of respondents stratified by primary DC role in the healthcare system (N = 1,455).

Variable, n (%)	Spine or NSMK care (N=1,030)	Subluxation detection and removal (N=208)	General primary care (N=109)	Overall
Likelihood to recommend DC school	(n=920)	(n=194)	(n=99)	(n=1,230)
Likely	68 (7.4%)	31 (16.0%)	10 (10.1%)	111 (9.0%)
Somewhat likely	156 (17.0%)	43 (22.2%)	21 (21.2%)	221 (18.0%)
Neutral	154 (16.7%)	30 (15.5%)	18 (18.2%)	202 (16.4%)
Somewhat unlikely	219 (23.8%)	31 (16.0%)	19 (19.2%)	275 (22.4%)
Unlikely	323 (35.1%)	59 (30.4%)	31 (31.3%)	421 (34.2%)
Likelihood to re-attend DC school today	(n=920)	(n=192)	(n=99)	(n=1,229)
Likely	84 (9.1%)	34 (17.7%)	15 (15.2%)	135 (11.0%)
Somewhat likely	132 (14.3%)	35 (18.2%)	14 (14.1%)	184 (15.0%)
Neutral	109 (11.8%)	17 (8.9%)	15 (15.2%)	141 (11.5%)
Somewhat unlikely	199 (21.6%)	25 (13.0%)	17 (17.2%)	243 (19.8%)
Unlikely	396 (43.0%)	81 (42.2%)	38 (38.4%)	526 (42.8%)
Career in chiropractic again or another field	(n=913)	(n=195)	(n=96)	(n=1,215)
I would pursue a career in chiropractic	299 (32.7%)	91 (46.7%)	31 (32.3%)	425 (35.0%)
I would NOT pursue a career in chiropractic	614 (67.3%)	104 (53.3%)	65 (67.7%)	790 (65.0%)
Other health care career	418 (68.8%, n=608)	59 (56.7%, n=104)	44 (68.8%, n=64)	532 (67.3%, n=790)
Non-health care career	190 (31.3%, n=608)	45 (43.3%, n=104)	20 (31.3%, n=64)	258 (32.7%, n=790)
Chiropractic training provided a positive ROI	(n=920)	(n=194)	(n=99)	(n=1,231)
Strongly agree	60 (6.5%)	30 (15.5%)	14 (14.1%)	104 (8.4%)
Agree	164 (17.8%)	32 (16.5%)	13 (13.1%)	211 (17.1%)
Neutral	203 (22.1%)	35 (18.0%)	20 (20.2%)	260 (21.1%)
Disagree	216 (23.5%)	39 (20.1%)	21 (21.2%)	281 (22.8%)
Strong disagree	277 (30.1%)	58 (30.0%)	31 (31.3%)	375 (30.5%)
Financial ROI of chiropractic training^a^	(n=921)	(n=195)	(n=98)	(n=1,231)
Very high ROI	26 (2.8%)	16 (8.2%)	3 (3.1%)	45 (3.7%)
High ROI	54 (5.9%)	10 (5.1%)	6 (6.1%)	70 (5.7%)
Moderate ROI	189 (20.5%)	37 (19.0%)	20 (20.4%)	250 (20.3%)
Low ROI	289 (31.4%)	51 (26.2%)	35 (35.7%)	380 (30.9%)
Very low ROI	363 (39.4%)	81 (41.5%)	34 (34.7%)	486 (39.5%)
Non-financial ROI of chiropractic training^b^	(n=918)	(n=194)	(n=97)	(n=1,226)
Very high ROI	87 (9.5%)	38 (19.6%)	12 (12.4%)	137 (11.2%)
High ROI	210 (22.9%)	44 (22.7%)	17 (17.5%)	275 (22.4%)
Moderate ROI	300 (32.7%)	57 (29.4%)	30 (30.9%)	392 (32.0%)
Low ROI	202 (22.0%)	29 (14.9%)	24 (24.7%)	259 (21.1%)
Very low ROI	119 (13.0%)	26 (13.4%)	14 (14.4%)	163 (13.3%)

SD: Standard deviation; IQR: Interquartile range; US: United States; DC: Doctor of Chiropractic; NMSK: neuromusculoskeletal; ROI: Return on investment; ^a^Respondents were asked to consider only financial factors such as current income, student loan debt, and the overall financial benefits experienced as a DC; ^b^Respondents were asked to consider only career advancement, professional satisfaction, work-life balance, professional flexibility, the ability to help others, and other non-monetary benefits or challenges.

More than half of respondents (56.6%) were unlikely or somewhat unlikely to recommend DCP training, whereas 16.4% of respondents were ambivalent to make a recommendation. Approximately half (53.3%) of respondents either disagreed or strongly disagreed that their DCP provided a positive ROI; whereas a quarter (25.6%) agreed or strongly agreed that their DCP provided a positive ROI. Over 70% of respondents indicated that DCP training has a low to very low financial ROI, with respondents in the subluxation-focused subgroup indicating slightly more favorable (13.3% high or very high ROI) outlooks compared to NMSK (8.7%) and primary care (9.2%) subgroups. Viewpoints of non-financial ROI were more positive universally, compared to financial ROI, among the three subgroups. The proportion of respondents reporting high to very high non-financial ROI was nearly quadruple financial ROI (33.6% compared to 9.3%, respectively).

### Cost of attendance estimates

For the identified 13 DCPs with publicly available total COA data, mean (SD) and median (Q1-Q3) estimates were $260,642 ($24,206) and $277,452 ($245,304-$280,050), respectively. By comparison, the mean (SD) and median (Q1-Q3) respondent estimates for total COA for attending chiropractic school in 2025 were $230,000 ($72,900) and $225,000 ($200,000-$250,000).

## Discussion

This is the first study to investigate self-reported student loan debt characteristics using survey data from a large cross-section of US DCs. This study is novel given its scale, and it is the first to report DCs’ student loan characteristics alongside demographics, practice patterns, repayment, and perceptions of DCP educational and career value. Survey respondents tended to reflect a younger demographic, a greater female representation, and a larger proportion of DCs who identified the best role for chiropractors in the healthcare system is as a NMSK expert, compared to other US DC workforce assessments.[34,44]

Findings in this study suggest DCs are burdened with considerable student loan debt that outpaces reported gross income – a motif not uncommon to US health professions more broadly. This is also congruent with a 2023 study using Bureau of Labor Statistics entry-level wage data in concert with published student loan data to calculate ability to repay student loans for various healthcare professions (including chiropractic) based on the amount of discretionary income afforded by their wages.[11] This model assumed higher earners have more money remaining after meeting basic necessities and thus can devote a larger proportion of their income towards paying down educational debt, compared to lower earners. Alarmingly, this study found that the DC profession was unable to meet recommended debt service ratios under any conventional repayment plan. The only instance in which DCs were able to meet salary-pegged benchmarks occurred under income-drive repayment plans which capped monthly student loan payments at 10% of income. Additionally, a DC degree failed to meet the financial value of a bachelor’s degree when accounting for monetary differences in earnings, debt, opportunity cost, and timing of wage initiation (i.e., shorter educational periods leading to sooner wages). The results from this study are consistent with these findings from previous work, especially high levels of debt, low reported income, broad use of income-driven repayment plans, and low perceptions of financial return on investment. This study’s findings also mirror the theme of high self-reported debt and low income published in the 2024 study of US chiropractic graduates who consulted with a student loan consultation firm.[16] From an economic perspective, the decision to pursue professional education can be conceptualized through the lens of human capital theory.[45,46] This theory frames higher education as an optimization process where the investment in skill development contributes to improvements in productivity. For the individual, this investment in forgone earnings during the period spent in training is made in anticipation of future benefits, such as higher earnings or increased job satisfaction. However, when educational debt becomes disproportionately large relative to expected income, it may produce “debt overhang”, a condition in which exceedingly greater debt burdens constrain career mobility, discourage risk-taking, and influence employment decisions. Further economic analysis, including detailed qualitative study, is warranted to assess whether the financial structure of chiropractic education aligns with sustainable career trajectories for graduates.

Both COA and income disillusionment may exist among US DCs. Respondents underestimated median COA by approximately $50,000, suggesting that respondents may have an incomplete, though not entirely absent, understanding of current DCP costs. Respondents’ estimation remained relatively stable as they moved farther from DCP graduation, suggesting misperception of total COA may exist irrespective of time since graduation. Concerningly, even respondents who recently graduated still underestimated the total COA by nearly $42,000 which may reflect a limited awareness of the true financial cost of their education. Nonetheless, since institutional COA estimates include projected living expenses, which can vary considerably given geography and personal choice, respondents’ estimates may differ legitimately from a chiropractic program’s budgetary projection without implying factual ignorance. The findings from this study also imply that most DCs are not earning $100,000 until they enter the second decade of their career. In contrast, DC student expectations regarding income suggest a minimum of a $100,000 annual income within the first few years of practice.[47,48] A majority of DC students (81%) anticipate annual income range of $40,001 to $100,000 within the first year of practice, over half of DC students (56%) expect to earn $100,001 to $500,000 within the first five years of practice, and the vast majority (87%) expect to earn at least $100,000 if not exceed $500,000 within 10 years of starting practice.[48] Responsible and transparent marketing of DC professional earnings and expectation management may foster a stronger sense of career fulfillment and a clearer understanding of their career path for future DCs.

Student loan debt held by survey respondents grew from graduation to survey completion, with a net increase of $55,000 in the median debt sum. This may be attributable to variations in repayment strategies rather than widespread shortfalls managing debt. It is probable that some DCs with less debt paid off loans more quickly, exiting the outstanding balance group, and resulting in a smaller respondent pool holding a higher average loan burden. Some DCs may also have voluntarily or involuntarily participated in general, administrative, or other forbearance or deferral programs with interest capitalization, leading to a growing loan balance. Given the prevalent use of income-drive repayment plans in this sample, it is also likely some DCs may have elected for participation in plans with longer durations in anticipation of student loan forgiveness while intentionally minimizing adjusted gross income and prioritizing allocations to tax-sheltered accounts to cover downstream tax obligations secondary to forgiveness. Similarly, DCs who actively or plan to participate in relief programs, such as PSLF, may purposely allow balances to grow, making minimum payments only, since loans will be forgiven after a set amount of service time irrespective of the total balance and without the future tax implications of income-driven repayment plan forgiveness. Given the latter two, it is possible some respondents have identified potentially effective management plans for their student loan debt despite the appearance of the contrary.

Both eligibility for and participation in student loan relief programs was exceedingly low among respondents. This finding is likely reflective of the limited number of relief-eligible job positions available to DCs rather than the limited uptake of relief opportunities since a similar proportion of individuals eligible for relief also participated. Slightly more respondents (n = 9) indicated participating in relief programs than were eligible based on their current professional position. This may indicate some respondents have previously participated in these programs but have since exited relief-eligible employment. Among all respondents, less than 10% indicated participating in PSLF. In contrast, in the medical profession, 46% of 2021 neurology-bound medical school graduates indicated pursuing PSLF between 2016 and 2020, and PSLF enrollment tripled from 7% to 22% for American Board of Family Medicine survey respondents.[49,50] Expanded employment opportunities in relief-eligible positions for DCs (akin to those of other healthcare professions) may help soften the burden of student loans and enhance perceptions of educational value.

In the literature, several studies have reported on DC job satisfaction globally, noting relatively high satisfaction, which contrasts with this study’s findings in a US-based population. Benyon et al. (2021), in an Australian DC population, reported overall high career satisfaction, especially for female chiropractors compared to males.[51] Konrad et al. (2004) identified US-based DC career satisfaction as relatively high and positively associated with intrinsic rewards of patient care, extrinsic rewards of compensation, and positive collegial relationships with other DCs.[52] This study’s novel approach to describe subgroups found modestly divergent perspectives regarding educational and career value among individuals self-identifying into one of three subgroups previously described.[34] Respondents in the subluxation-focused subgroup were more likely to recommend DCP training, re-attend their DCP, and pursue a career in chiropractic again compared to those in the NMSK or primary care subgroups. While speculative, this may be indicative of a deeper sense of professional identity, or “calling” to the profession, as well as greater ideological alignment within this subgroup. However, despite the subluxation-focused subgroup having more positive perceptions compared to the NMSK and primary care subgroups, the majority of respondents in each professional subgroup still held overall negative perceptions of their DCP training. Regardless of professional subgroup, the majority of respondents would not pursue a career in chiropractic again, which may be related to lower perceptions of educational ROI, particularly in the financial domain. While DCs may not perceive high financial ROI, non-financial benefits such as work-life balance, flexibility, and the ability to help others appear to provide recognizable value. Nearly 1 in 5 respondents would choose a career outside of healthcare altogether, likely attributable to factors related to intent to leave all health professions (e.g., burnout, renumeration, career opportunities, job satisfaction, leadership, disengagement) alongside potential chiropractic-specific factors (e.g., physical demand, negative media portrayal/public opinion).[53,54]

The findings from this study, as an initial descriptive assessment, are concerning and indicative of a general, and likely systemic, trend regarding the sustainability of the US chiropractic profession given a rising graduate debt burden and tenuous wages, suspected to be driven at least in part by low reimbursement for chiropractic services.[55,56] In the context of health professions education, value is represented by the benefits one incurs (e.g., earnings, satisfaction, flexibility) divided by the cost of securing those benefits (e.g., tuition/fees, opportunity costs). Given the ever-changing landscape of higher education economics (e.g., limiting total federal and professional program student loan allotments, sunsetting of Graduate PLUS loans, fluctuations in available research funding), chiropractic profession stakeholders are encouraged to engage in solution-oriented collaboration to co-create actionable steps to address affordability, quality, and value of DCPs to trainees and ensure long-term viability of the profession. Relevant stakeholders include prospective DCP students, DCP graduates, DCPs, the clinical industry, accrediting organizations, regulatory entities, advocacy and professional trade organizations, and patients/public. These findings can serve to anchor data-driven dialogue and provide an empirical springboard for innovation and strategic planning among these groups to deliver systemic change.

### Future directions

Further investigations of DC student loan burden are necessary to inform strategies to mitigate loan burden (i.e., reduce cost of training or increase gross income) and manage borrower’s expectations. A national, organized effort to routinely assess recent DCP graduate’s income, student loan debt, loan relief participation and career satisfaction, akin to other healthcare professions, should be considered.[7,9,57] However, individual or shared ownership, roles, and responsibilities of this effort across key organizations in US DCP education should be defined. Areas for future research include evaluating the DC’s debt-to-income ratio, loan burden, and return-on-investment associations for a variety of DC subgroup characteristics such as: (1) US census region of practice, (2) cash versus insurance based collections, (3) chiropractic professional subgroups, (4) minoritized groups, (5) post-graduate training (i.e., residency, fellowship), (6) access and eligibility to debt relief programs, (7) employment status (i.e., private, group, hospital), and (8) years of practice experience.[51] Understanding these relationships could help identify relevant modifiable factors to inform targeted interventions and/or policy change. Additionally, a better understanding of the maximum supportable debt limit for a DC degree based on commensurate debt loads, discretionary income, and repayment options is warranted and could be modeled after prior work.[58] Finally, qualitative investigations into the lived experience of DC borrowers managing the burden of student loan debt are justified to capture the holistic (e.g., emotional and social) impacts, including their motivations for specific repayment strategies (e.g., income-driven repayment plan with or without targeting forgiveness), financial planning, and the influence on major life decisions (e.g., family planning, purchasing a house).

### Limitations and strengths

This study possesses limitations inherent to a web-based, cross-sectional survey using non-probability sampling. The adopted method of distribution may have introduced coverage errors if individuals lacked membership to trade organizations or social media, had outdated or limited alumnus contact information, or low interaction levels with these communication mediums. We attempted to address this selection bias with multiple avenues and time points of contact. While our multi-channel recruitment strategy, including snowball sampling, aimed to maximize reach, the resulting sample may over-represent individuals with higher levels of professional engagement or those with a particular interest in the financial topics surveyed. Because the survey was distributed via open links on social media and through third-party listservs, the total number of individuals reached (the true denominator) remains unknown and precludes calculation of an accurate response rate. Consequently, a completion rate derived from survey link clicks provides only a proxy for engagement. This likely serves as the lower-bound estimate of true participation among those who intended to complete the survey.

Additionally, the survey was conducted entirely online which may have limited participation to digitally literate individuals. While we distributed the survey through nationally representative groups, we cannot be certain that the findings are not skewed by geographical location. Moreover, this study’s findings are only applicable to US-trained DCs and may not represent the ROI or other financial characteristics of ex-US chiropractors or chiropractic education on the global stage. We intentionally did not report or stratify results by DCP to maintain sensitivity to the national burden of student loans and to not uniquely single out any individual DCP, reporting DCP-specific details only from publicly available information. We did not assess how participants discovered the survey, which could impact future recruitment efforts for follow-up studies. Since respondents self-selected into the sample, we cannot be certain what motivated some DCs to respond to the survey while others did not. However, we do suspect that we captured more responses from DCs with current student loans than those without, thus potentially biasing the study sample and results towards higher debt burdens. Given the anonymous nature of the survey and lack of personalized links, we also could not prevent multiple participation by single individuals. We also were unable to account for respondents who had multiple loans and repayment strategies associated with a single degree, and we did not attempt to discern the portion of total student debt specifically allocable to DC training. We did not perform validity evaluations of the survey constructs beyond pilot testing for face and content validity; therefore, interpretation of questions and responses may not have been consistent across all respondents. The survey topics may have been considered controversial or emotionally driven thus eliciting a social desirability bias.

While we used statistical methods and subject-matter expertise to identify and exclude suspected outliers or erroneous values, it is possible these represent genuine responses. Our approach of pairing statistical detection of outliers using GESD with human judgment was likely sufficient to identify and exclude outliers given the anticipated number of outliers was small and the data distribution supported its assumptions. A validation step demonstrated that our reported findings were robust to these exclusions, as the overall distribution and measures of central tendency remained consistent regardless of whether the outlier values were retained or removed.

This study was conducted during a period when the SAVE repayment program was blocked by the US court system and many borrowers were placed into a forced forbearance repayment status which may have impacted reporting on current payment amounts and accurate reporting of accrued interest. Given the timing of this study, its findings should thus be interpreted within the broader context of SAVE. We also conducted this survey at the beginning of 2025 and collected income information based on the 2023 tax year, as we suspected some respondents may not have yet had 2024 tax year information available. This makes it possible that some income responses were underestimated, especially for recent graduates who may have started working after 2023 but before survey completion.

Despite its limitations, this study has several important strengths. To the authors’ knowledge, this is the first survey of US DCs regarding student loan debt, perceived value of DCP, and career fulfilment. Its robust sample (n = 1,455) represents 21 DCPs spanning a broad array of years since graduation, suggesting a diverse range of respondent experiences. The study’s demographics skewed slightly younger than other demographic profiles of the US chiropractic profession; however, since student loans may be more likely to impact early career DCs, this appears reasonable. For item design where responses required numerical values (e.g., total loan amount at present, loan interest rate, monthly payments), we applied logical constraints to field entry (e.g., numerical ranges) which may have helped to limit interpretive variance. We maintained respondent anonymity throughout to minimize social desirability bias. While we present trends in average financial metrics by years since graduation without weighting for sample size, thus potentially reflecting regression to the mean, and without controlling for other confounding variables, we believe assessment of the overall trend to be adequate and informative in this exploratory analysis. Future work can consider regression analyses that account for individual-level data while controlling for relevant covariates.

## Conclusions

Overall, US DCs carry a student loan burden that often exceeds their annual gross income. Findings of this study are commensurate with prior studies investigating chiropractic educational debt. Even so, the findings are likely not altogether unique to the chiropractic profession but rather are representative of larger challenges faced by many modern US health professions. This study is novel given its scale and is the first to describe loan characteristics, demographics, practice patterns, repayment, and perceptions of educational value. Few DCs participate in loan relief programs while many participate in income-drive repayment, suggesting most are responsible for full repayment or standard forgiveness. The perception of current DCP cost is underestimated, and most DCs would not pursue a career in chiropractic given the repeat opportunity, choosing either a career outside of healthcare or another healthcare discipline. The majority of DCs do not find that their DCP training provided a positive ROI, especially financially. Perceptions of general career path selection and financial ROI were consistently negative across US DC professional subgroups. Innovation and collaboration among various stakeholders in DC higher education are likely necessary to optimize long-term viability and financial sustainability of the profession. Further work is indicated to probe associations among DC’s student loan debt variables and to determine manageable debt loads for the profession.

## Supporting information

S1 Appendix Checklist for Reporting of Survey Studies (CROSS). (PDF)

S2 Appendix Survey Instrument. (PDF)

S3 Appendix Doctor of chiropractic program total cost of attendance. (PDF)

S4 Appendix Additional degrees attained by respondents (N = 1,455). (PDF)

S5 Appendix Degree repayment plans of respondents (N = 1,455). (PDF)

S6 Appendix Years in repayment on degree(s) by respondents (N = 1,455). (PDF)

## Abbreviations

CCE-USA: Council on Chiropractic Education – United States
US: United States
DC: Doctor of Chiropractic
DCP: Doctor of Chiropractic program
ACA: American Chiropractic Association
ICA: International Chiropractic Association
REDCap: Research Electronic Data Capture
VA: United States Department of Veterans Affairs
DOD: Department of Defense
NMSK: Spine/Neuromusculoskeletal
COA: Cost of Attendance
ROI: Return on Investment
PSLF: Public Service Loan Forgiveness
IHS: Indian Health Service; NIH, National Institutes of Health
NHSC: National Health Service Corps
HRSA: Health Resources and Services Administration
SAVE: Saving on a Valuable Education
