# Supplementary material for: A survey of student loan burden among United States Chiropractors: Insights on debt, relief, and educational value
Source: PLoS One. 2026 Apr 13;21(4):e0347127. doi: 10.1371/journal.pone.0347127 (PMC13075670; doi:10.1371/journal.pone.0347127)
Supplement: S2 Appendix — (PDF) [file pone.0347127.s002.pdf]

Student Loan Burden among US Chiropractors

Thank you for your interest in this survey. Please read the following information regarding your participation.

SUMMARY

We are asking you to join a research study. The purpose of this research study is to understand the characteristics of US chiropractors' student loan debt and explore associations between student loans and other variables. Examples of the types of questions you will encounter include: "Approximately how much did you owe in total student loans when you graduated from your Doctor of Chiropractic program?", "Since the first time you made a student loan payment, how long have you been making payments?", and "Given the value of your education and occupation, how likely would you be to attend chiropractic school again?". Your involvement will require 10-15 minutes. The results from this survey may be presented in educational settings, at professional conferences, and published in professional journals.

ELIGIBILITY

Eligible participants include graduates from a US-based, CCE-accredited Doctor of Chiropractic program. Those who are current students, have never attended a CCE-accredited Doctor of Chiropractic program, or have attended a non-US chiropractic program are ineligible to participate in this survey.

PROCEDURES

This electronic survey will be completed using Yale University's Research Electronic Data Capture (REDCap) system. The survey includes questions about your demographic and educational background, your practice perspectives, your loan characteristics, your eligibility for both federal/state or employer-sponsored student loan relief programs, and your progress towards loan forgiveness (if applicable). You will also be asked about your likelihood to recommend a DC career, the perceived value of your education, and your probability to pursue a DC career again.

Several questions will require knowledge of the types of student loans you have or have had in the past, the total student loan amount borrowed, total student loan amount owed currently, your current student loan payment, your current student loan interest rate, and any forgiveness programs you have received or are presently pursuing. You may wish to collect this information before or during this survey.

RISKS

We do not anticipate any physical risks from taking part in this study. You will be asked questions about your demographics, student loans, and professional career decisions. Some may be uneasy about responding to such questions. This is an anonymous survey; no personally identifiable information will be captured. Where appropriate, cell suppression techniques will be used to report low frequency counts. Survey data will be securely maintained with access limited to the research team and minimal risk of inappropriate access by someone who is not a member of the study team.

BENEFITS

There are no direct benefits for an individual taking part in this study. You may like helping to contribute to research on student loan debt in the chiropractic profession. You may enjoy talking about your career in chiropractic.

PARTICIPATION IS VOLUNTARY

Your participation is voluntary. You are free to withdraw your participation in this study at any time. If you do not want to continue, you can simply leave this website. If you feel uncomfortable with a question, you may skip it or withdraw from the study entirely. If you decide to quit at any time prior to completing the questionnaire and you do not click on the "Submit" button at the end of the survey, your answers will not be recorded.

CONFIDENTIALITY

Your responses will be anonymous and confidential. We will not know your IP address when you respond to this online survey.

PAYMENT

No payment or other compensation is being offered for participation.

CONTACT INFORMATION

If you have questions about your rights as a study subject, or you want to make sure this is a valid Yale University study, you may contact the Yale Institutional Review Board at (203) 785-4688 or email hrpp@yale.edu. If you have questions, complaints, or concerns about the study, or if you would like to obtain information or offer input, you may contact Dr. Brian Coleman (brian.coleman@yale.edu).

By beginning this survey, I acknowledge that I have read the information above, confirmed my eligibility, and agree to participate in this research, with the knowledge that I am free to withdraw my participation at any time without penalty.

- ◯ I agree to participate
- ◯ I decline to participate

Eligibility Confirmation

Are you a graduate from a US-based, CCE-accredited Doctor of Chiropractic program?

◯ Yes
◯ No

Demographics and Practice Characteristics

What is your current age?

____
(Please enter a whole number (minimum: 18).)

How many years has it been since you graduated from a Doctor of Chiropractic program?

____
(Please enter a whole number. If you graduated less than 1 year ago, enter 0.)

Approximately what was your gross income for the 2023 tax year?

____
(Please enter a whole number without symbols.)

Are you male or female?

◯ Male ◯ Female ◯ Decline to answer

How would you best describe your race/ethnicity?

◯ Hispanic ◯ Native Hawaiian / Pacific Islander ◯ non-Hispanic White ◯ non-Hispanic Black ◯ non-Hispanic Asian ◯ Multiracial ◯ Hispanic Other ◯ non-Hispanic Other ◯ Other

Are you currently practicing chiropractic in the United States?

◯ Yes
◯ No

Have you ever practiced chiropractic in the United States?

◯ Yes
◯ No

Which of the following best classifies your current area of primary work (defined as work accounting for greatest percentage of professional effort)?

◯ Clinical practice ◯ Administrative ◯ Education ◯ Research ◯ Retired ◯ Other

In what clinical setting, if any, have you most recently spent time practicing chiropractic in?

☐ Solo practice ☐ Group practice (DCs only) ☐ Franchised practice ☐ Multi-disciplinary practice (DCs and non-DC clinicians) ☐ Veterans Affairs (VA)/Department of Defense (DoD) ☐ Hospital system (non-VA/DoD) ☐ Post-Graduate Residency or Fellowship ☐ Other Academic Healthcare Setting ☐ Other ☐ Never in clinical practice

Which United States Doctor of Chiropractic program conferred your Doctor of Chiropractic degree?	◯ Cleveland University-Kansas City College of Chiropractic ◯ D'Youville College ◯ Keiser University ◯ Life Chiropractic College West ◯ Life University ◯ Logan University ◯ National University of Health Sciences, Florida Campus ◯ National University of Health Sciences, Illinois Campus ◯ Northeast College of Health Sciences (formerly New York Chiropractic College) ◯ Northwestern Health Sciences University ◯ Palmer College of Chiropractic, Main Campus ◯ Palmer College of Chiropractic, West Campus ◯ Palmer College of Chiropractic, Florida Campus ◯ Parker University ◯ Sherman College of Chiropractic ◯ Southern California University of Health Sciences ◯ Texas Chiropractic College ◯ Universidad Central del Caribe ◯ University of Bridgeport, College of Chiropractic ◯ University of Western States, College of Chiropractic ◯ Other
Please enter which "Other" Doctor of Chiropractic program conferred your Doctor of Chiropractic degree:	__________________________________
What do you feel is the one best role for chiropractors in the greater healthcare system?	◯ Subluxation detection and removal ◯ General primary care ◯ Spine or neuromusculoskeletal care

Educational Background

Have you completed any other post-secondary education degree(s) in addition to your Doctor of Chiropractic? Select all that apply.	☐ Associate degree(s) ☐ Bachelor's degree(s) ☐ Master's or graduate degree(s) ☐ Other doctoral or professional degree(s) ☐ None

Select when you completed the following degree program(s) in comparison to your Doctor of Chiropractic program (DCP)?

Associate degree(s)	◯ Prior to matriculating into DCP<br>◯ While concurrently enrolled in DCP (during your DC degree program or conferred degree alongside DC degree)<br>◯ After graduating from DCP
Bachelor' s degree(s)	◯ Prior to matriculating into DCP<br>◯ While concurrently enrolled in DCP (during your DC degree program or conferred degree alongside DC degree)<br>◯ After graduating from DCP
Master's or graduate degree(s)	◯ Prior to matriculating into DCP<br>◯ While concurrently enrolled in DCP (during your DC degree program or conferred degree alongside DC degree)<br>◯ After graduating from DCP
Other doctoral or professional degree(s)	◯ Prior to matriculating into DCP<br>◯ While concurrently enrolled in DCP (during your DC degree program or conferred degree alongside DC degree)<br>◯ After graduating from DCP

Loan Characteristics

The following questions are in US Dollars and refer to federal and private student loans obtained during all levels of education (undergraduate and graduate).

At the time you graduated from your Doctor of Chiropractic program, did you have any student loan debt (federal or private, from any level of education)?

◯ Yes ◯ No

Approximately how much did you owe in total student loans when you graduated from your Doctor of Chiropractic program?

____
(Please enter a whole number without symbols.)

As of today, do you currently have any student loan debt (federal or private, from any level of education)?

◯ Yes ◯ No

As of today, approximately how much do you owe in total student loans?

____
(Please enter a whole number without symbols.)

What is the approximate interest rate (%) across your student loans? If you are currently in a period of interest rate change due to administrative or other forbearance, please enter the approximate interest rate when not in forbearance. Please enter the percentage rate, rounded to one decimal place (e.g., 6.5).

____
(Please enter the percentage value, rounded to one decimal place.)

As of today, approximately what is the monthly payment amount of your student loans?

____
(Please enter a whole number without symbols.)

Which of the following student loans do you currently hold? Select all that apply.

☐ Subsidized Federal Loan
☐ Unsubsidized Federal Loan
☐ Grad PLUS Loan ☐ Parent PLUS Loan
☐ Consolidation Loan ☐ Perkins Loan
☐ Health Education Assistance Loan (HEAL)
☐ Private Loan ☐ Other

Select the current status of your student loans for each degree you've previously identified attaining. If you completed a degree but did not have student loans associated with that degree, select No Loans.

Associate degree(s)	◯ Grace period<br>◯ Repayment<br>◯ Deferment<br>◯ Forbearance<br>◯ Delinquent<br>◯ Default<br>◯ Paid in full<br>◯ No Loans
Bachelor' s degree(s)	◯ Grace period<br>◯ Repayment<br>◯ Deferment<br>◯ Forbearance<br>◯ Delinquent<br>◯ Default<br>◯ Paid in full<br>◯ No Loans
Master's or graduate degree(s)	◯ Grace period<br>◯ Repayment<br>◯ Deferment<br>◯ Forbearance<br>◯ Delinquent<br>◯ Default<br>◯ Paid in full<br>◯ No Loans
Doctor of Chiropractic degree	◯ Grace period<br>◯ Repayment<br>◯ Deferment<br>◯ Forbearance<br>◯ Delinquent<br>◯ Default<br>◯ Paid in full<br>◯ No Loans
Other doctoral or professional degree(s)	◯ Grace period<br>◯ Repayment<br>◯ Deferment<br>◯ Forbearance<br>◯ Delinquent<br>◯ Default<br>◯ Paid in full<br>◯ No Loans

Additional student loan characteristics

Are you currently in administrative forbearance due to Saving on a valuable Education (SAVE) litigation?	◯ Yes ◯ No ◯ Unsure ◯ N/A<br>(The Saving on a Valuable Education (SAVE) is the newest income-driven repayment plan, which was announced August 22, 2023, by the Biden-Harris administration. Since then, a federal court has issued a nationwide injunction which prevents the US Department of Education from implementing certain component of SAVE. As a result, SAVE borrowers have been placed in administrative forbearance by their student loan servicers and are not required to make monthly payments while in this forbearance at this time. (Information as of Jan 29, 2025))
Since the first time you made a student loan payment, for how many years have you made, or did you make, payments on loans resulting from your associate degree(s)? If you have been making payments less than 1 year, select 0.	______________________<br>(Please enter a whole number without symbols.)
Since the first time you made a student loan payment, for how many years have you made, or did you make, payments on loans resulting from your bachelor's degree(s)? If you have been making payments less than 1 year, select 0.	______________________<br>(Please enter a whole number without symbols.)
Since the first time you made a student loan payment, for how many years have you made, or did you make, payments on loans resulting from your master's or graduate degree(s)? If you have been making payments less than 1 year, select 0.	______________________<br>(Please enter a whole number without symbols.)
Since the first time you made a student loan payment, for how many years have you made, or did you make, payments on loans resulting from your Doctor of Chiropractic degree? If you have been making payments less than 1 year, select 0.	______________________<br>(Please enter a whole number without symbols.)
Since the first time you made a student loan payment, for how many years have you made, or did you make, payments on loans resulting from your other doctoral or professional degree(s)? If you have been making payments less than 1 year, select 0.	______________________<br>(Please enter a whole number without symbols.)

Select which repayment plan that you are currently using or most recently used for your student loans.

If you are currently in administrative forbearance due to SAVE litigation, select which repayment plan you were using prior to SAVE related administrative forbearance.

If you have multiple student loans with different repayment plans, please select the option that corresponds to the repayment plan for the majority of your student loan debt.

Associate degree(s)	◯ Standard<br>◯ Graduated<br>◯ Extended<br>◯ Income-Driven<br>◯ Other
Bachelor' s degree(s)	◯ Standard<br>◯ Graduated<br>◯ Extended<br>◯ Income-Driven<br>◯ Other
Master's or graduate degree(s)	◯ Standard<br>◯ Graduated<br>◯ Extended<br>◯ Income-Driven<br>◯ Other
Doctor of Chiropractic degree	◯ Standard<br>◯ Graduated<br>◯ Extended<br>◯ Income-Driven<br>◯ Other
Other doctoral or professional degree(s)	◯ Standard<br>◯ Graduated<br>◯ Extended<br>◯ Income-Driven<br>◯ Other

Loan Relief Programs

Regardless of whether you currently have student loan debt, are you currently employed in a professional position that is eligible for any student loan relief (forgiveness, discharge, or repayment) programs?	◯ Yes ◯ No ◯ Unsure
Which of the following programs does the professional position in which you are employed qualify for? Select all that apply.	☐ Public Service Loan Forgiveness Program ☐ Indian Health Service Loan Repayment Program ☐ National Health Service Corps Loan Repayment Program ☐ Faculty Loan Repayment Program ☐ National Institutes of Health Loan Repayment Program ☐ Employer sponsored Loan Repayment or Forgiveness Program ☐ State specific loan relief program ☐ Other
Have you participated in or are you currently participating in any student loan relief (forgiveness, discharge, or repayment) programs?	◯ Yes ◯ No ◯ Unsure
Which of the following programs have you participated in? Select all that apply.	☐ Public Service Loan Forgiveness Program ☐ Indian Health Service Loan Repayment Program ☐ National Health Service Corps Loan Repayment Program ☐ Faculty Loan Repayment Program ☐ National Institutes of Health Loan Repayment Program ☐ Employer sponsored Loan Repayment or Forgiveness Program ☐ State specific loan relief program ☐ Other ☐ None
What is the total number of qualifying payments you have currently made towards Public Service Loan Forgiveness? Do not include payments still requiring employer certification.	______________________ (Please enter a whole number without symbols.)
What is the total number of all potentially eligible payments you currently made towards Public Service Loan Forgiveness? Include both qualifying and payments requiring employer certification.	______________________ (Please enter a whole number without symbols.)
Have you ever sought out employment opportunities based on their eligibility for student loan relief (forgiveness or repayment) programs?	◯ Yes ◯ No

Career Value and Fulfillment

What do you estimate as the average total cost of attendance for US chiropractic school at present (as of 2025), including tuition, fees, class supplies, and living expenses for the entire duration of the program?

____
(Please enter a whole number without symbols.)

How likely are you to recommend chiropractic school to someone interested in the field?

- ◯ Likely
- ◯ Somewhat likely
- ◯ Neutral
- ◯ Somewhat unlikely
- ◯ Unlikely

Given the value of your education and occupation, how likely would you be to attend chiropractic school again if you were making the decision today?

- ◯ Likely
- ◯ Somewhat likely
- ◯ Neutral
- ◯ Somewhat unlikely
- ◯ Unlikely

Thinking about your career path and the value you place on your professional training and experience, if you were starting your professional training today, would you pursue a career in chiropractic again?

- ◯ I would pursue a career in chiropractic
- ◯ I would NOT pursue a career in chiropractic

Knowing you wouldn't choose to pursue a career in chiropractic again, if you were starting your professional training today, would you still pursue a career in health care?

- ◯ I would pursue career in health care other than chiropractic
- ◯ I would pursue a career outside of health care

Please rate your agreement with the following statement: Chiropractic training provided a positive return on my investment.

- ◯ Strongly Agree
- ◯ Agree
- ◯ Neutral
- ◯ Disagree
- ◯ Strongly Disagree

Thinking specifically about the financial aspects of your chiropractic education, how would you rate the return on your investment of your chiropractic training? Please consider only financial factors such as your current income, student loan debt, and the overall financial benefits you've experienced as a chiropractor.

- ◯ Very High Return on Investment
- ◯ High Return on Investment
- ◯ Moderate Return on Investment
- ◯ Low Return on Investment
- ◯ Very Low Return on Investment

Beyond the financial aspects of your chiropractic education, how would you rate the overall return on investment of your chiropractic training? Consider factors such as career advancement, professional satisfaction, work-life balance, professional flexibility, the ability to help others, and other non-monetary benefits or challenges you may have experienced.

- ◯ Very High Return on Investment
- ◯ High Return on Investment
- ◯ Moderate Return on Investment
- ◯ Low Return on Investment
- ◯ Very Low Return on Investment
