# Supplementary material for: A survey of student loan burden among United States Chiropractors: Insights on debt, relief, and educational value
Source: PLoS One. 2026 Apr 13;21(4):e0347127. doi: 10.1371/journal.pone.0347127 (PMC13075670; doi:10.1371/journal.pone.0347127)
Supplement: S3 Appendix — (PDF) [file pone.0347127.s003.pdf]

S3 Appendix. Doctor of chiropractic program total cost of attendance.

Most Doctor of Chiropractic Programs (DCPs) provide publicly accessible cost of attendance (COA) calculators to help prospective and matriculated students and families understand the aggregate cost of degree programs, including tuition, fees, housing, and meal plans, before any financial aid is applied. Programs develop these estimates based on data from the most recent years' Consumer Expenditure Survey produced by the US Bureau of Labor Statistics. (Data as of 10/22/2024)

United States Doctor of Chiropractic Program Institution	2024-25 Cost of Attendance
Cleveland University – Kansas City College of Chiropractic	$284,480
D'Youville College	$239,141
Keiser University	-
Life University	$246,516
Life Chiropractic College West	$245,304
Logan University	$280,050
National University of Health Sciences	-
Northeast College of Health Sciences	$216,583
Northwestern Health Sciences University	$283,177
Palmer College of Chiropractic	$224,555
Parker University	$279,600
Sherman College of Chiropractic	$278,040
Southern California University of Health Sciences	$281,209
Texas Chiropractic College	$252,238
University of Bridgeport School of Chiropractic	-
Universidad Central del Caribe	-
University of Western States	$277,452
Mean (SD)	$260,642 ($24,207)
Median (Q1-Q3)	$277,452 ($245,304-$280,050)

SD: Standard deviation; Q1-Q3: 1st quartile-3rd quartile
