# Supplementary material for: A survey of student loan burden among United States Chiropractors: Insights on debt, relief, and educational value
Source: PLoS One. 2026 Apr 13;21(4):e0347127. doi: 10.1371/journal.pone.0347127 (PMC13075670; doi:10.1371/journal.pone.0347127)
Supplement: S4 Appendix — (PDF) [file pone.0347127.s004.pdf]

S4 Appendix. Additional degrees attained by respondents (N=1,455)[a]

Degree	n (%)
Associate degrees	137 (9.4%)
Prior to DCP	135
During DCP	1
After DCP	1
Bachelor’s degrees	1034 (71.1%)
Prior to DCP	831
During DCP	181
After DCP	13
Master’s or other graduate degrees	200 (13.7%)
Prior to DCP	49
During DCP	83
After DCP	66
Other Doctoral or Professional degrees	74 (5.1%)
Prior to DCP	3
During DCP	4
After DCP	66
No additional degrees	178 (12.2%)

DCP: Doctor of Chiropractic program; [a]Non-exclusive groups, some missing secondary responses
