# Supplementary material for: A survey of student loan burden among United States Chiropractors: Insights on debt, relief, and educational value
Source: PLoS One. 2026 Apr 13;21(4):e0347127. doi: 10.1371/journal.pone.0347127 (PMC13075670; doi:10.1371/journal.pone.0347127)
Supplement: S5 Appendix — (PDF) [file pone.0347127.s005.pdf]

S5 Appendix. Degree repayment plans of respondents (N=1,455)

Degree	n (%)
Associate degrees (n=102)	
Standard	14 (13.7%)
Graduated	2 (2.0%)
Extended	0 (0.0%)
Income-Driven	39 (38.2%)
Other	47 (46.1%)
Bachelor's degrees (n=576)	
Standard	130 (22.6%)
Graduated	18 (3.1%)
Extended	7 (1.2%)
Income-Driven	357 (62.0%)
Other	64 (11.1%)
Master's or other graduate degrees (n=118)	
Standard	15 (12.7%)
Graduated	4 (3.4%)
Extended	0 (0.0%)
Income-Driven	90 (76.3%)
Other	9 (7.6%)
Doctor of Chiropractic degree (n=1,034)	
Standard	123 (11.9%)
Graduated	30 (2.9%)
Extended	15 (1.5%)
Income-Driven	837 (80.9%)
Other	29 (2.8%)
Other Doctoral or Professional degrees (n=17)	
Standard	1 (5.9%)
Graduated	0 (0.0%)
Extended	0 (0.0%)
Income-Driven	9 (52.9%)
Other	7 (41.2%)
