# Supplementary material for: A survey of student loan burden among United States Chiropractors: Insights on debt, relief, and educational value
Source: PLoS One. 2026 Apr 13;21(4):e0347127. doi: 10.1371/journal.pone.0347127 (PMC13075670; doi:10.1371/journal.pone.0347127)
Supplement: S6 Appendix — (PDF) [file pone.0347127.s006.pdf]

S6 Appendix. Years in repayment on degree(s) by respondents (N=1,455)

Degree	Years
Associate degrees (n=68)	
Mean (SD)	5.16 (6.25)
Median (Q1-Q3)	2.50 (0-10)
Min, Max	0, 23.0
Bachelor's degrees (n=678)	
Mean (SD)	6.63 (6.61)
Median (Q1-Q3)	5.00 (1-10)
Min, Max	0, 30.0
Master's or other graduate degrees (n=139)	
Mean (SD)	4.50 (5.02)
Median (Q1-Q3)	3.00 (0-7)
Min, Max	0, 25.0
Doctor of Chiropractic degree (n=1212)	
Mean (SD)	8.14 (7.07)
Median (Q1-Q3)	7.00 (2-13)
Min, Max	0, 40.0
Other Doctoral or Professional degrees (n=29)	
Mean (SD)	3.10 (5.60)
Median (Q1-Q3)	0 (0-5)
Min, Max	0, 25.0

SD: Standard deviation; Q1-Q3: 1st quartile-3rd quartile
